# Linking growth dynamics and intra-annual density fluctuations to late-summer precipitation in humid subtropical China

**DOI:** 10.3389/fpls.2025.1568882

**Published:** 2025-06-25

**Authors:** Chunsong Wang, Zhuangpeng Zheng, Jiani Gao, Feifei Zhou, Sergio Rossi, Keyan Fang

**Affiliations:** ^1^ Institute of Geography, Fujian Normal University, Fuzhou, China; ^2^ Key Laboratory of Humid Subtropical Eco-Geographical Process (Ministry of Education), College of Geographical Sciences, Fujian Normal University, Fuzhou, China; ^3^ School of Tourismand Historical Culture, Zhaoqing University, Zhaoqing, China; ^4^ Département des Sciences Fondamentales, Université du Québec à Chicoutimi, Chicoutimi, QC, Canada

**Keywords:** xylogenesis, wood anatomy, autumn growth peak, L-IADFs, extreme climate events, Southeastern China

## Abstract

Global warming has intensified extreme rainfall events and prolonged droughts, significantly impacting tree growth and wood formation. This study investigates the effects of late-summer precipitation variability on the intra-annual growth dynamics of *Cunninghamia lanceolata* and *Cryptomeria fortunei* in humid subtropical China. Microcores were collected from 12 trees at 7–10 days intervals between March and December from 2021 to 2023 in the Gushan Mountains. Typically, high temperatures and rainfall deficits in July induce cambial dormancy, while subsequent rainfall in August and September reactivates growth, resulting in a bimodal growth pattern. However, in 2022, an unprecedented drought (August–October rainfall 77% below average) shortened the growing season, causing an early cessation of growth and a rare unimodal growth pattern. In contrast, persistent rainfall in 2023 accelerated cell enlargement to 7 μm d^-^¹ and significantly increased latewood intra-annual density fluctuations (L-IADFs). Notably, despite abundant late-summer rainfall in 2021, L-IADFs did not form, indicating a nonlinear and inconsistent relationship between rainfall and L-IADFs. These findings highlight the critical role of late-summer precipitation variability in shaping tree growth patterns and wood density in southeastern China. Given the expected increase in precipitation variability under climate change, regional forest ecosystems may become more vulnerable. This study provides valuable insights for forest management strategies to enhance resilience and mitigate climate-related risks.

## 1 Introduction

In the recent decades, global warming has led to significant disruptions in seasonal precipitation patterns, intensifying extreme rainfall events and prolonging dry periods (Iles et al., 2024; IPCC, 2021; Feng et al., 2013). These climatic shifts have profound implications for forest ecosystems, as they affect the dynamics of wood formation processes, contributing to an increased tree mortality and forest degradation (Feldman et al., 2024; Zhou et al., 2024; Allen et al., 2010). Wood formation is sensitive to fluctuations in temperature and precipitation, which directly influence xylem development and lead to significant variations in growth patterns (Rathgeber et al., 2016; Rossi et al., 2016). Understanding how these climate-induced changes affect wood formation is critical for predicting the resilience and dynamics of forest ecosystems under global warming.

In temperate and alpine regions, xylem growth typically follows a unimodal pattern, with earlywood formation in spring and latewood development in late summer, followed by growth cessation in autumn (Huang et al., 2023; Rossi et al., 2006b). However, in regions with more complex climates, such as the Mediterranean and humid subtropical areas, trees often experience summer droughts, leading to distinct growth dynamics and anatomical patterns in xylem formation (Zlobin, 2022; Vieira et al., 2019). Summer droughts in these regions can induce bimodal growth patterns, with growth peaks in both spring and autumn (Häusser et al., 2023; Wang C. S. et al., 2023; Jiang et al., 2021). Autumn growth peaks are generally associated with the resumption of precipitation, which stimulates cambial activity and promotes latewood cell enlargement (Niccoli et al., 2024; Zalloni et al., 2016).

Autumn growth peaks often correspond to the formation of latewood intra-annual density fluctuations (L-IADFs) in the Mediterranean, characterized by earlywood-like cells with large lumens and thin walls within latewood. These fluctuations are typically triggered by abrupt changes in moisture availability (Balzano et al., 2019). In humid subtropical regions, where there is a pronounced shift from summer drought to autumn rainfall (Ma et al., 2021; Xu et al., 2019), climatic fluctuations drive adaptive changes in xylem width and structure, significantly influencing wood formation dynamics (Häusser et al., 2023; Zhang et al., 2020). As indicators of environmental variability, L-IADFs provide insights into how trees respond to climatic stressors such as droughts and sudden changes in moisture, reflecting their growth strategies and adaptive capacity (Niccoli et al., 2024; Gao et al., 2021a). These wood structure traits highlight the ability of trees to adjust their architecture for efficient water transport and mechanical support—key factors for forest resilience in a changing climate (Zalloni et al., 2016). Understanding these traits is crucial for predicting the long-term evolution of forests under future climate change.

Despite extensive research on the frequency of IADFs in tree rings, the underlying physiological mechanisms driving their formation remain poorly understood. Most studies have focused on documenting the occurrence and frequency of IADFs, with limited attention given to the physiological processes and environmental drivers behind their formation (Wang S.J. et al., 2023; De Micco et al., 2016). This knowledge gap hinders our understanding of the factors influencing L-IADFs formation, especially in regions with significant precipitation variability. Recent advances in xylem kinetics offer promising approaches to bridge this gap by analyzing the timing and rates of xylem cell formation alongside anatomical changes. This integrated approach can provide deeper insights into the processes of cell division, enlargement, and wall thickening (Cuny et al., 2014), helping to identify critical periods when climatic factors, such as precipitation, promote or inhibit xylem formation. For example, Gao et al. (2021b) demonstrated that water scarcity alters both the timing of cell division and the rate of cell enlargement, which ultimately influences xylem morphology. These findings emphasize the importance of linking wood anatomy and xylem dynamics to better understand L-IADF formation and its response to climatic drivers.

This study focuses on two conifer species in humid subtropical China: *Cunninghamia lanceolata* and *Cryptomeria fortunei*. These species were chosen due to their widespread distribution and ecological importance in the region. We aim to investigate the anatomical characteristics of xylem cells and analyze the dynamics of xylem formation in response to variability in late-summer precipitation. Using microcores collected over three consecutive years, we explore how late-summer precipitation influences xylem cell development, with particular attention to autumn growth peaks and L-IADF formation. We hypothesize that: (1) the amount and timing of late-summer precipitation control the intensity and duration of the autumn growth peak; (2) continuous heavy precipitation promotes cell enlargement and L-IADFs formation; and (3) a growth peak in autumn does not always correspond to L-IADFs formation.

## 2 Materials and methods

### 2.1 Study area

**Figure 1:**
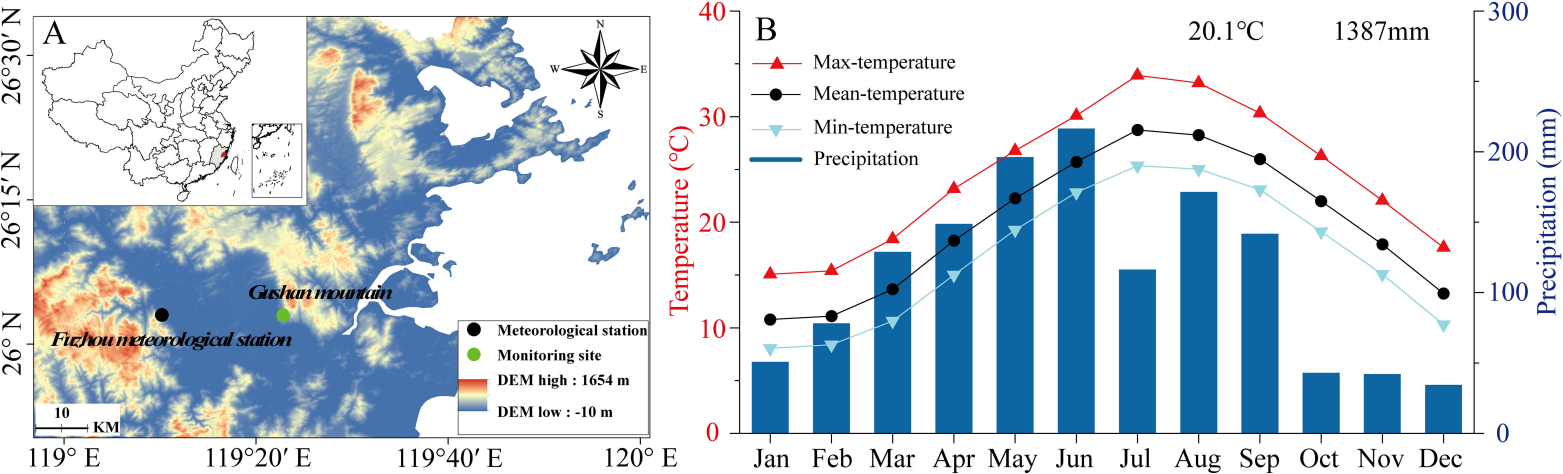
Location of the study site **(A)** and climate in the Fuzhou meteorological station during 1951-2020 **(B)**. The upper right of the diagram shows an annual temperature and total precipitation.

This study was conducted in the Gushan Mountains, a humid subtropical region of southeastern China (26.05°N, 119.38°E, 450 m a.s.l.), strongly influenced by the East Asian monsoon (**Figure 1A**). The forest canopy is predominantly composed of *Cunninghamia lanceolata (C. lanceolata)* and *Cryptomeria fortune (C. fortunei)*, and contains minor species of *Pinus massoniana* and *Schima superba* (Zheng et al., 2021). The area experiences a humid subtropical climate, with a mean annual temperature of 20.1°C (1951–2020). July experiences the highest monthly temperature of 34.29°C, while January is the coldest month at 8.16°C. The area receives an average annual precipitation of 1387 mm, 72% of which occurs between March and August. A typical summer drought occurs in July (**Figure 1B**). Daily climate data, including precipitation, temperature, soil moisture, and relative humidity, were collected from the Fuzhou Meteorological Station (26.08°N, 119.28°E, 85 m a.s.l.), located 11.2 km away from the study site. Data were accessed from the China Meteorological Administration (https://weather.cma.cn/).

### 2.2 Tree selection and sample collection

Seven healthy *C. lanceolata* and five *C. fortunei* trees were selected for monitoring stem radial growth. The selected trees were of similar dominance and had well-developed crowns. The *C. lanceolata* trees had an average diameter at breast height (DBH) of 27.4 ± 2.7 cm (mean ± SD), a height of 7.9 ± 1.0 m, and an estimated age of 35 ± 2 years (mean ± SD). The *C. fortunei* trees had an average DBH of 28.4 ± 4.8 cm (mean ± SD), a height of 12.8 ± 1.5 m, and an estimated age of 31.6 ± 1.4 years (mean ± SD). Microcores were collected at 7–10-day intervals during the growing seasons (March to December) from 2021 to 2023 at breast height (1.3 m) using a Trephor (Rossi et al., 2006a). Samples were immediately preserved in a 9:1 solution of 70% alcohol and acetic acid. Each core, 2 mm in diameter and 2–3 cm in length, contained two intact tree rings and adjacent phloem. Sampling points were systematically arranged around the trunk to minimize interference, maintaining a minimum spacing of 5 cm between successive samples. In the laboratory, samples were dehydrated using an alcohol gradient (70%, 90%, 95%, and 100%), cleared with limonene, and embedded in paraffin. Transverse sections (8 μm thick) were cut with a rotary microtome (HistoCore BIOCUT, LEICA, Wetzlar, Germany) and stained with safranin and Astra blue (Rossi et al., 2003; Rossi et al., 2006b).

### 2.3 Xylem formation dynamics

Transverse sections were examined under visible and polarized light using a 200× optical microscope (DM750P, LEICA, Wetzlar, Germany) to identify xylem cells at different differentiation stages. Cambium cells were recognized as flattened cells with thin walls, while enlarging cells appeared larger with irregular diameters. Wall-thickening cells were identified by their birefringence under polarized light, and mature cells were characterized by fully developed structures, which stained red with safranin (Li et al., 2017). The onset of xylem differentiation was marked by the appearance of at least one row of enlarging cells in spring. Xylem cell division cessation in autumn was defined by the absence of enlarging cells, and differentiation was considered complete when no wall-thickening cells were present (Rossi et al., 2006b). Cell counts at each developmental stage were recorded during every sampling interval and correlated with the day of the year (DOY) to create a timeline of xylem development.

### 2.4 Quantification of xylem anatomical parameters

Digital images of xylem sections were captured at 200× magnification using a microscope with an integrated camera (MC190 HD, LEICA, Wetzlar, Germany). Anatomical parameters, such as radial lumen diameter (LD), cell wall thickness (CWT), and tracheid diameter (TD, where TD = LD + 2×CWT), were measured along five radial files using ImageJ software (National Institutes of Health, Bethesda, MD, USA). Mature cells were classified as earlywood (4×CWT/LD < 1) or latewood (4×CWT/LD ≥ 1) based on Mork’s criterion (Denne, 1989). To assess intra-annual variability, each growth ring was divided into 10 equal radial sections. Anatomical parameters for all cells within each section were averaged, enabling segment-wise analysis of growth ring anatomical features. This approach facilitated the identification of intra-annual variations in tracheid characteristics (Silvestro et al., 2023).

### 2.5 Data analysis and statistics

To account for variations in growth rates across different trunk directions (Wang C. S. et al., 2023), xylem cell counts were standardized using the following formula:


(1) nci=ncmi×rwm/rws

where *nc_i_* is the standardized number of xylem cells, *ncm_i_* is the measured number of cells, *rw_m_* is the mean width of preceding tree rings across all samples, and *rw_s_* is the width of preceding tree rings for the specific sample (Rossi et al., 2006b).

The Gompertz function was applied to analyze xylem formation kinetics, estimating the duration of cell enlargement (d_E_) and wall thickening (d_WT_) phases. Rates of cell enlargement (r_E_=TD/d_E_) and wall thickening (r_WT_=2×CWT/d_WT_) were calculated from the anatomical parameters of the percentage of the growth ring (Gao et al., 2021b; Palombo et al., 2018).

Vapor Pressure Deficit (VPD) was calculated using air temperature (T) and relative humidity (RH) as follows:


(2) $$VPD=0.6108\times e^{\left(17.27T/(237.3+T))\times (1-RH\right)}$$

Monthly climate indices and key phenological timings (2021–2023) were compared using ANOVA following normality tests. Statistical analyses were conducted using IBM SPSS Statistics (version 25.0, SPSS Inc., Chicago, USA).

## 3 Results

### 3.1 Weather conditions during the study years

From March to November in 2021 to 2023, the climate deviated significantly from the 1980–2020 baseline. During the growing seasons, average temperatures were 0.86°C higher in 2021, 0.68°C higher in 2022, and 0.77°C higher in 2023. Precipitation exhibited substantial variability: an increase of 117.54 mm in 2021, a decrease of 173.54 mm in 2022, and a 50% increase (659.32 mm) in 2023. Precipitation fluctuations were more pronounced from July to November. Compared to the 1980–2020 average, rainfall increased by 179.86 mm in 2021, decreased by 299.95 mm in 2022, and surged by 747.55 mm in 2023 (**Table 1**). A severe drought occurred between August and October 2022, leading to extremely low soil moisture and a high VPD. In contrast, 2023 experienced two consecutive typhoons, which brought significant rainfall, alleviating the effects of high temperatures and drought. A similar pattern was observed in 2021, although with less rainfall over a shorter period. Consequently, the moisture conditions during August–September differed significantly (*p* < 0.05) across the three study years (**Table 2**).

**Table 1:** Difference from average value during 1980–2020 at Fuzhou meteorological station during 2021–2023 growing season.

Year	Growing season		Early growing season		Lare growing season	
	Temperature (°C)	Precipitation (mm)	Temperature (°C)	Precipitation (mm)	Temperature (°C)	Precipitation (mm)
2021	0.86	117.54	1.01	-62.31	0.73	179.86
2022	0.68	-173.54	-0.04	126.41	1.25	-299.95
2023	0.77	659.32	0.82	-88.22	0.73	747.55

Growing season (March-November), early growing season (March-June), late growing season (July-November).

**Table 2:** Monthly average temperature, soil moisture and VPD of late growing season during 2021-2023.

Index	Year	July	August	September	October	November
Temperature	2021	30.03 ^a^	28.57 ^a^	28.89 ^a^	24.63 ^a^	18.26 ^a^
(°C)	2022	29.81 ^a^	30.76 ^b^	28.33 ^a^	23.81 ^a^	20.19 ^b^
	2023	29.89 ^a^	28.78 ^a^	28.16 ^a^	23.85 ^a^	19.71 ^b^
Soil moisture	2021	35.42 ^a^	38.59 ^a^	34.51 ^a^	35.2 ^a^	35.84 ^a^
(Kg/m^2^)	2022	34.89 ^a^	30.64 ^b^	29.68 ^b^	27.3 ^b^	35.02 ^ab^
	2023	35.87 ^a^	38.01 ^a^	36.99 ^c^	34.6 ^a^	34.2 ^b^
VPD	2021	1.03 ^a^	0.51 ^a^	0.88 ^a^	0.59 ^a^	0.38 ^a^
(kPa)	2022	1.17 ^a^	1.37 ^b^	1.14 ^b^	0.82 ^b^	0.38 ^a^
	2023	1.02 ^a^	0.64 ^a^	0.61 ^c^	0.55 ^a^	0.54 ^b^

Different letters indicate significant differences between years (*p* < 0.05).

### 3.2 Xylem formation dynamics

**Figure 2:**
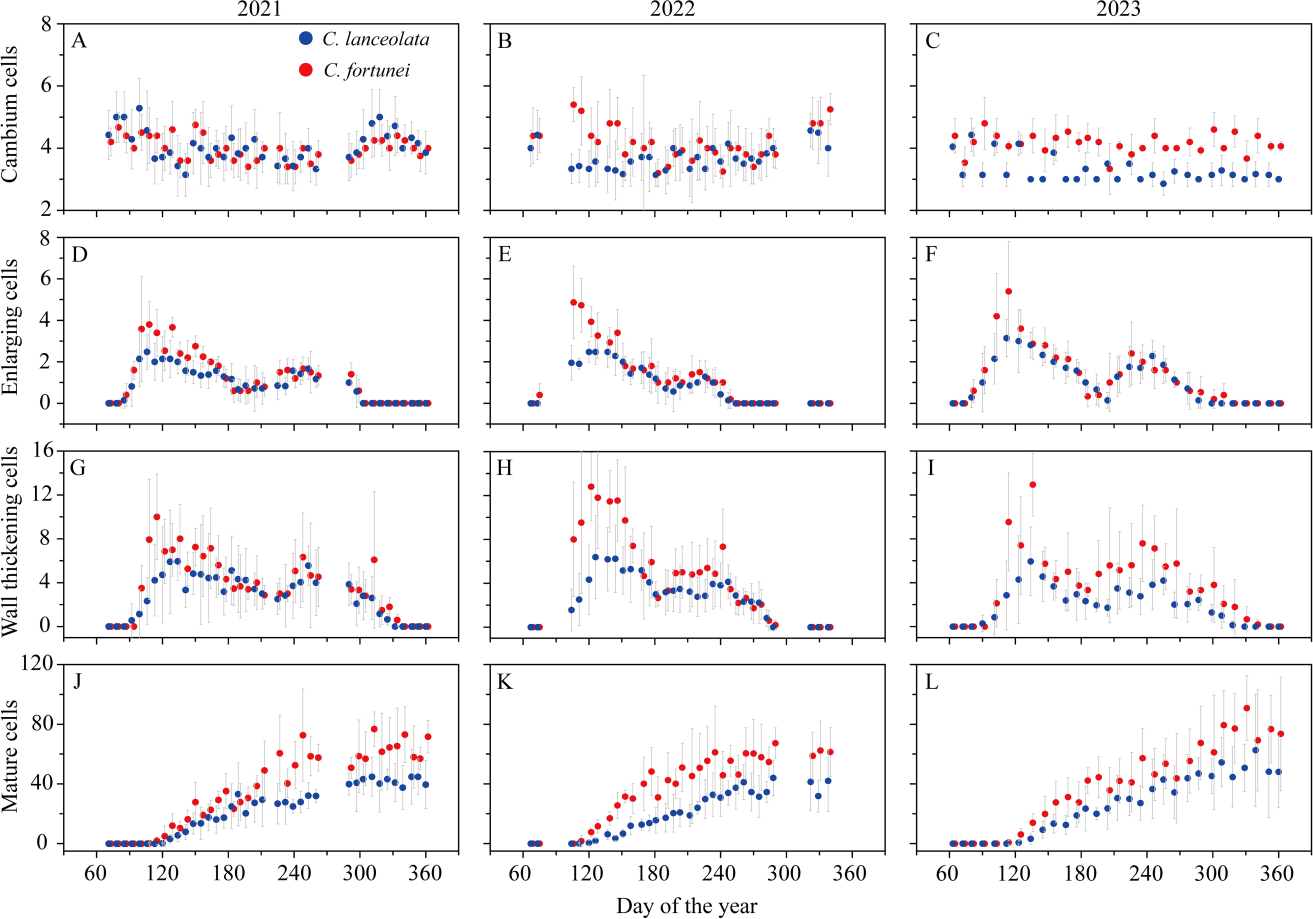
Number of cambium **(A–C)**, enlarging **(D–F)**, wall thickening **(G–I)**, and mature **(J–L)** cells of *C. lanceolata* (blue) and *C. fortunei* (red) during 2021-2023 at the Gushan Mountain. Error bars indicate the standard deviation between trees.

Xylem formation dynamics exhibited notable variation across the years, though the overall patterns remained consistent for both *C. lanceolata* and *C. fortunei*. During the growing season, cambial cell numbers exhibited slight fluctuations, ranging from 3 to 5 in *C. lanceolata* and 3 to 6 in *C. fortunei* over the three years (2021–2023). Notable inter-annual variability was observed in the dynamics of cell enlargement. In 2021, both species exhibited a bimodal growth pattern, with a primary peak in spring (around DOY 120) and a smaller second peak in autumn (around DOY 240), most pronounced in *C. fortunei*. In 2023, the bimodal pattern was even more pronounced, with a distinct autumn growth peak. In contrast, 2022 followed a unimodal pattern, with a single peak in spring. Cell wall thickening dynamics followed a similar trend to cell enlargement, though the peak occurred later due to the time required for wall thickening. Cell maturation followed an S-shaped curve, with the highest cell production recorded in 2023 and the lowest in 2022 (**Figure 2**).

### 3.3 Cambial phenology

**Figure 3:**
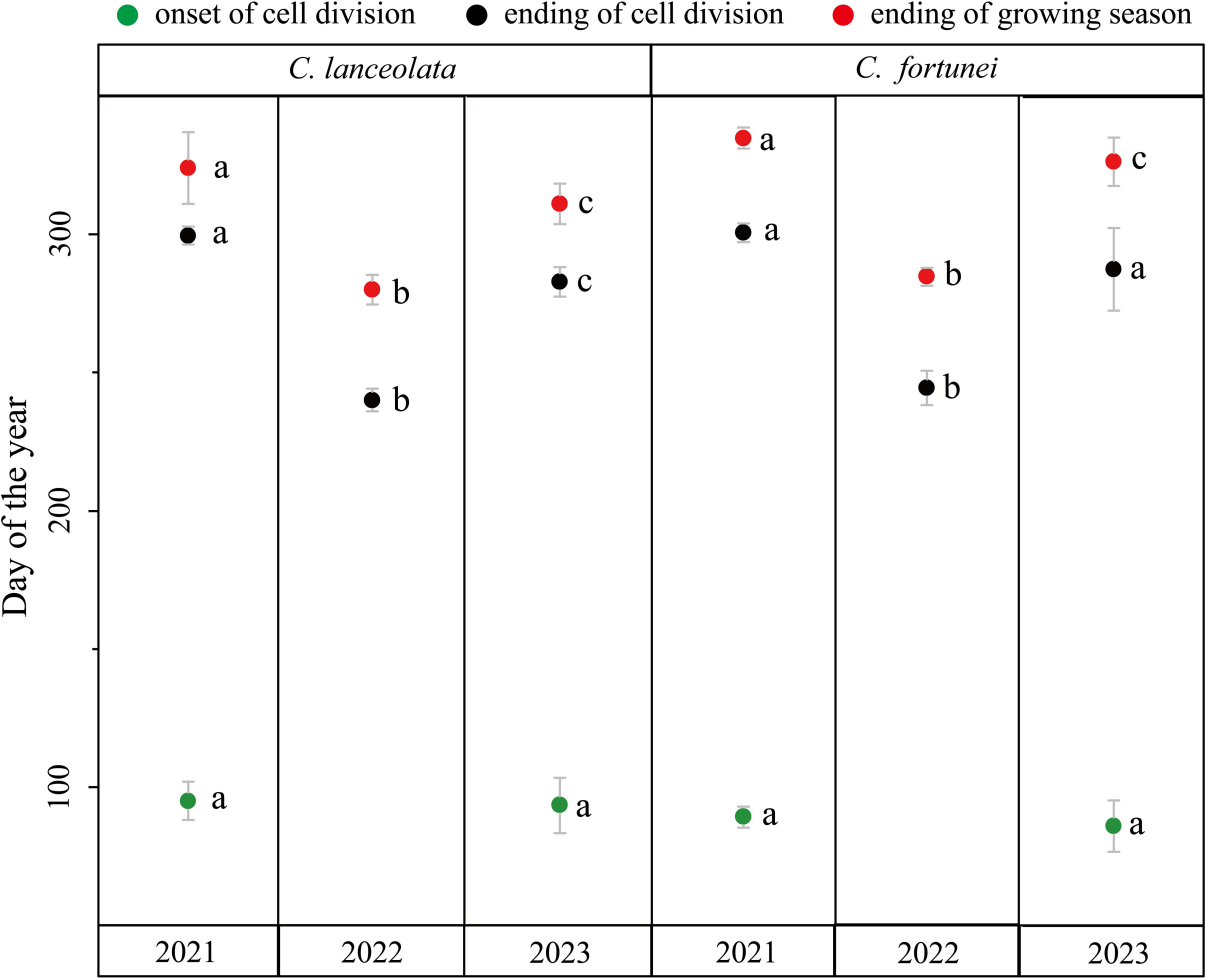
Cambial phenology of *C. lanceolata* and *C. fortunei* during the 2021–2023 reported as the timing of onset and ending of xylem cell division, and the ending of the growing season. Different letters indicate significant differences between years (p<0.05). Error bars indicate the standard deviation.

**Figure 4:**
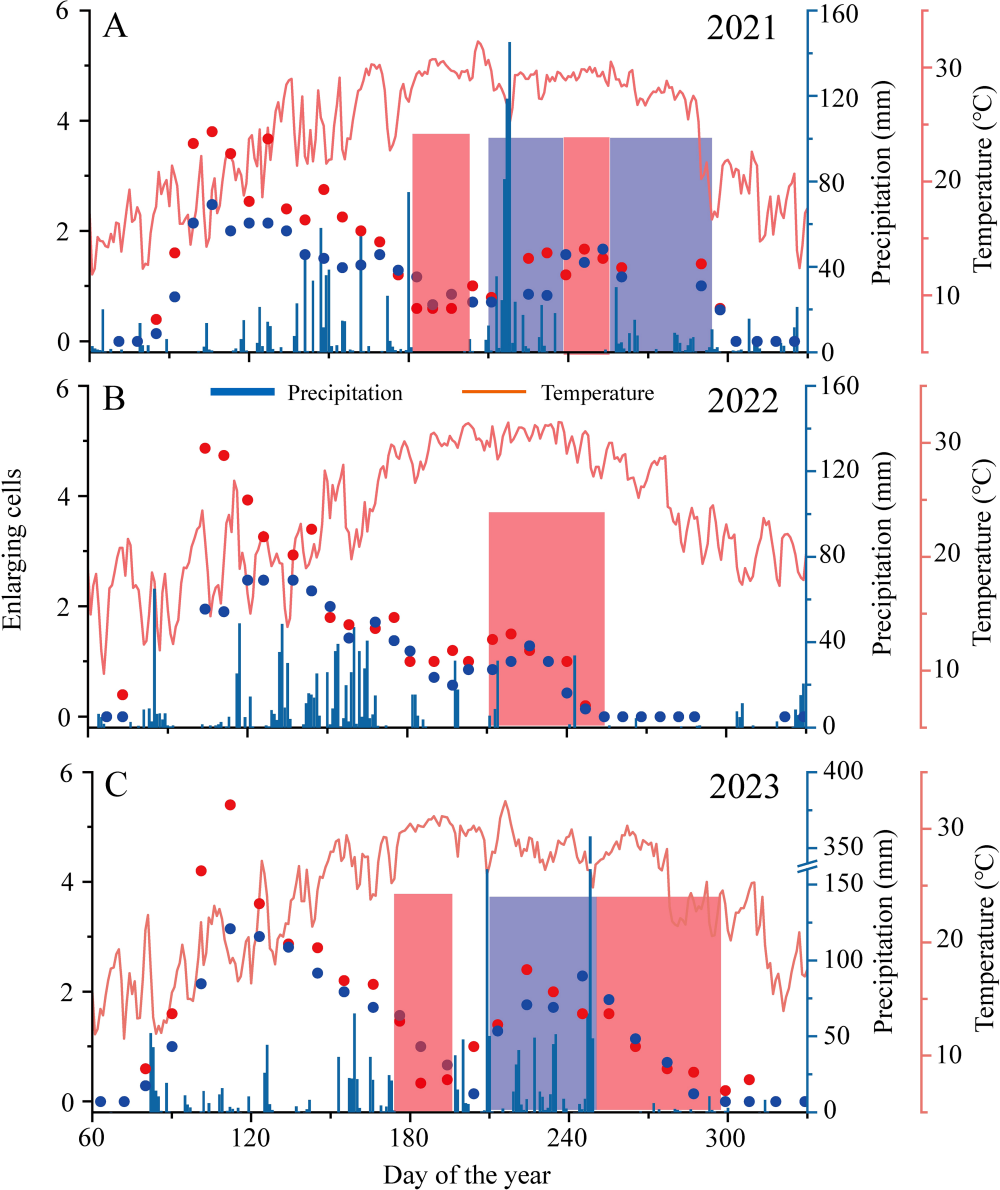
Weather conditions at the sampling site and enlarging cells dynamics of *C. lanceolata* (blue) and *C. fortunei* (red) during the growing season of 2021 **(A)**, 2022 **(B)**, and 2023 **(C)**. The shaded area represents the abnormal moisture period. The blue shading indicates a wetter period, while the red shading indicates a drier period.

Significant inter-annual variations in xylem formation, particularly during late summer and autumn, resulted in marked differences in cambial phenology. Due to road closures to Gushan Mountain from March to April 2022, data on the initiation of cambial activity were unavailable. In 2021, cambial activity began on DOY 95 ± 7 for *C. lanceolata* and on DOY 89 ± 7 for *C. fortunei*. In 2023, activity began on DOY 93 ± 10 for *C. lanceolata* and on DOY 86 ± 9 for *C. fortunei*, with no significant difference in onset timing between the two years. However, the cessation of xylem cell division and the end of the growing season showed considerable variation across years, except for *C. fortunei* in 2021 and 2023, where no significant differences were observed (**Figure 3**). Drought periods of varying intensity during late summer and autumn in 2022 and 2023 significantly advanced the timing of xylem cell division cessation (**Figure 4**). For *C. lanceolata*, cell division ceased on DOY 300 ± 3 in 2021, occurring 60 days earlier in 2022 and 17 days earlier in 2023. The end of the growing season shifted from DOY 324 ± 13 in 2021, advancing 44 days in 2022 and 13 days in 2023. For *C. fortunei*, the trend was consistent, with cell division ending 56 days earlier in 2022 and 17 days earlier in 2023 compared to DOY 300 ± 3 in 2021. The end of the growing season advanced by 51 days in 2022 and 8 days in 2023, relative to DOY 335 ± 4 in 2021 (**Figure 3**).

### 3.4 Xylem anatomical traits

**Figure 5:**
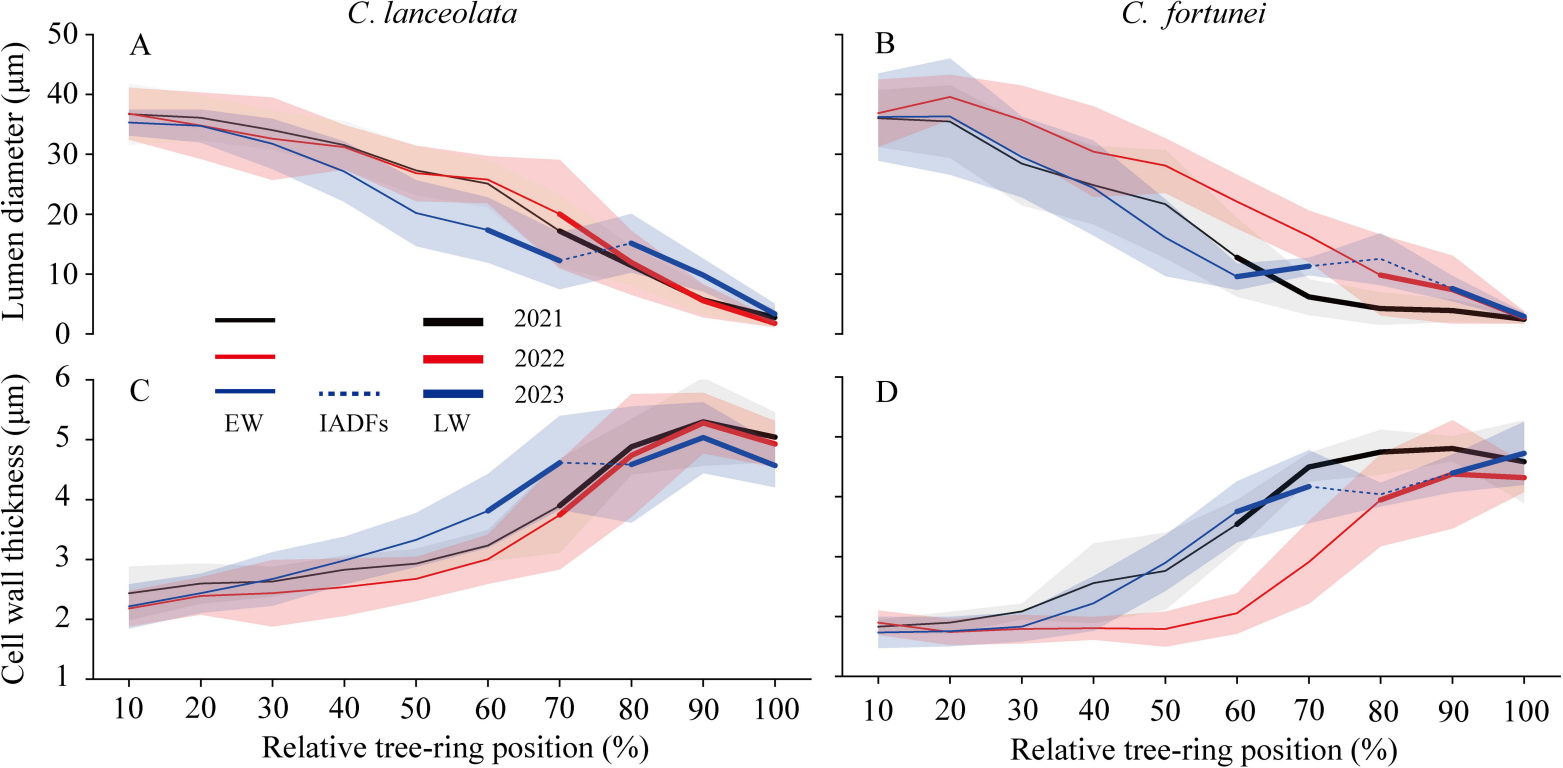
The relative position across the growth ring for the radial lumen diameter **(A, B)** and the cell wall thickness **(C, D)** of *C. lanceolata* and *C. fortunei* during 2021-2023. Thin lines indicate earlywood cells, dotted lines indicate IADFs, and thick lines indicate latewood cells. Lines show the mean values and shaded areas show one standard deviation. EW, earlywood cell; LW, latewood.

Xylem cell anatomy exhibited significant inter- and intra-annual variability. In *C. lanceolata*, the lumen diameter ranged from 2.45 μm to 39.6 μm. It consistently decreased from 10% of the growth ring, except for a modest enlargement at 80% across the tree ring in 2023 (**Figure 5A**). Cell wall thickness increased, starting at 2.18 μm and reaching a maximum of 5.30 μm at about 90% of the way across the tree ring, with a slight reduction at the final part. However, in 2023, a slight decrease in wall thickness was observed at 80% of the growth ring (**Figure 5C**). For *C. fortunei*, the lumen diameter was largest at 20% of the growth ring across all three years (35.87, 39.57, 36.32 μm, respectively), after which it consistently decreased (2.44, 2.75, 2.91 μm, respectively). However, by 2023, cells had expanded at 70%–80% of the tree ring (**Figure 5B**). The cell wall thickness ranged from 1.73 μm to 4.81 μm. In 2023, thickness initially increased, followed by a temporary decrease at 80% of the growth ring before rising again (**Figure 5D**).

### 3.5 Kinetics of cell development

**Figure 6:**
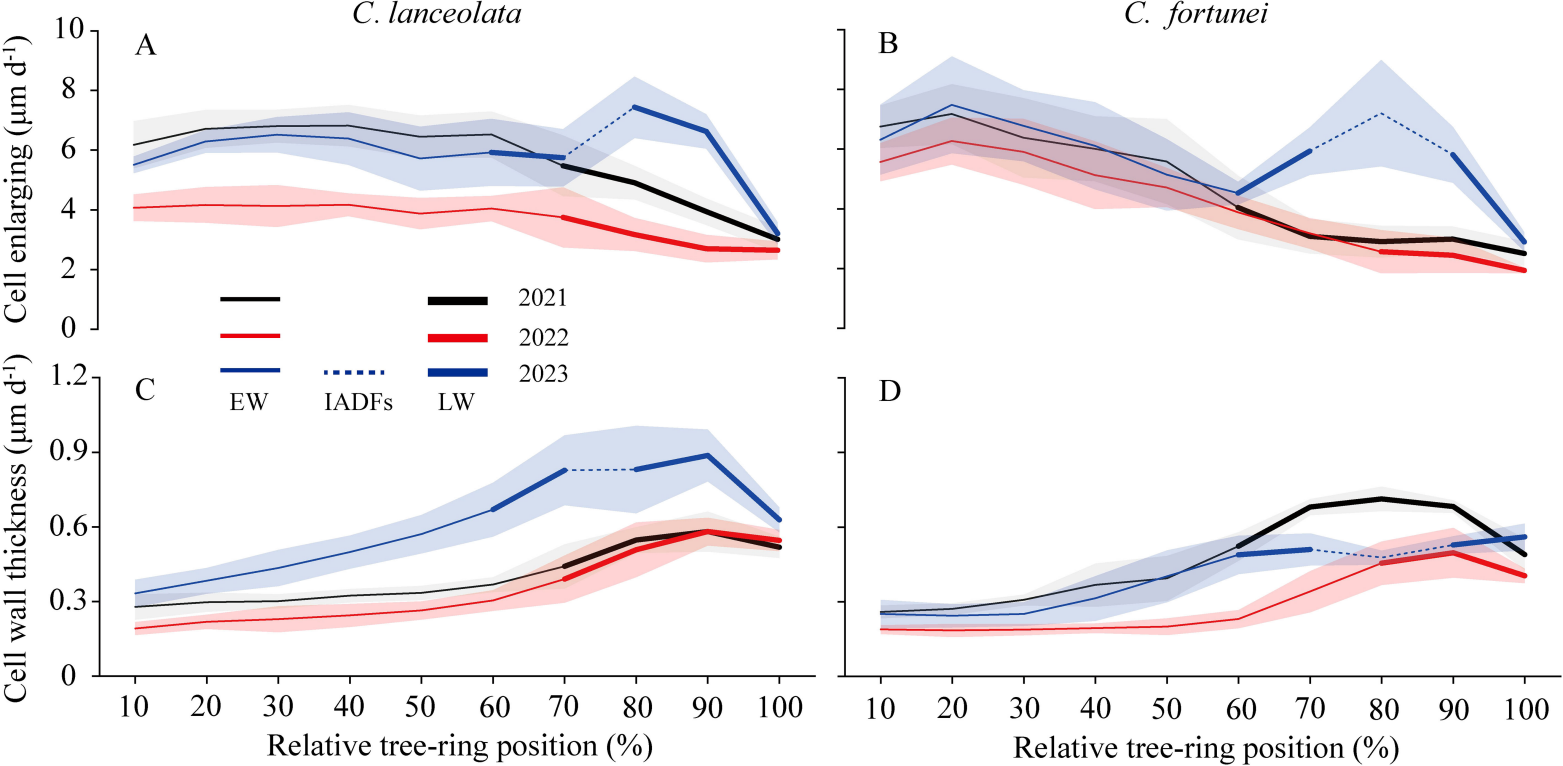
The relative position across the growth ring for the rate of cell enlarging **(A, B)** and the rate of cell wall thickening **(C, D)** of *C. lanceolata* and *C. fortunei* during 2021-2023. Thin lines indicate earlywood cells, dotted lines indicate IADFs, and thick lines indicate latewood cells. EW, earlywood cell; LW, latewood.

In 2023, heavy late-summer rainfall promoted the latewood cell enlargement rate (r_E_), which doubled, thus forming L-IADFs. For *C. lanceolata*, r_E_ remained consistently high during earlywood development (averaging 6.42, 4.03, and 6.06 μm d^-^¹), while the cell wall thickening rate (r_WT_) gradually increased (averaging 0.33, 0.26, and 0.48 μm d^-^¹). During latewood formation, r_E_ declined sharply as r_WT_ accelerated, peaking at 90% of the growth ring before tapering off. Notably, in 2023, an abrupt rise in r_E_ was observed at 80% of the growth ring (7.44 μm d^-^¹), resulting in a larger lumen diameter and relatively thinner cell wall compared to other latewood cells (**Figures 6A, C**). For *C. fortunei*, 20% of the growth ring exhibited the highest r_E_ (7.18, 6.27, and 7.49 μm d^-^¹, respectively), which then declined sharply. During latewood formation, r_E_ typically decreased gradually. However, a significant increase in r_E_ was observed between 70% and 80% of the tree ring in 2023. The rate increased by 125% (compared to the average of the other two years), reaching 6.57 μm d^-^¹. This resulted in cells with enlarged lumens and thinner walls, resembling earlywood cells. The r_WT_ steadily increased from 10% to 90% of the tree ring, followed by a decrease in the final part. However, in 2023, r_WT_ decelerated at 80%–90% of the tree ring, while it continued to rise in the final part (**Figures 6B, D**).

## 4 Discussion

### 4.1 Growth dynamics under different precipitation regimes

The bimodal growth pattern, characterized by two distinct phases of xylem cell production, has been reported in both Mediterranean and humid subtropical regions (Zheng et al., 2021; Huang et al., 2018; Camarero et al., 2010). This pattern is primarily driven by seasonal fluctuations in moisture availability, particularly during spring and autumn (Jiang et al., 2021; Garcia-Forner et al., 2019). In our study, both *C. lanceolata* and *C. fortunei* exhibited distinct growth peaks in spring and autumn, with growth stagnating during the dry summer months (**Figure 2**). This bimodal growth pattern aligns with the typical rainfall regime of humid subtropical China, suggesting that bimodal xylem production is a characteristic feature of conifer species in this region.

Inter-annual variability in precipitation from 2021 to 2023 significantly influenced xylem division during the late growing season. In 2023, reduced precipitation towards the end of the growing season led to an earlier cessation of cell division (**Figure 3**). However, abundant late-summer rainfall in the same year triggered a more pronounced autumn growth peak. In contrast, the lack of late-summer precipitation in 2022 resulted in early cessation of xylem growth, eliminating the autumn growth peak (**Figure 4B**). These inter-annual variations highlight the adaptability of trees to changing environmental conditions. Specifically, under drought stress, trees tend to adopt a conservative growth strategy to maintain physiological stability, while under wetter conditions, they accelerate growth to maximize resource allocation (Tumajer et al., 2021). In three years of observations, we found no significant inter-annual difference in the onset of xylem cell division, suggesting that the previous year’s conditions had little direct impact on the current year’s xylem phenology (**Figure 3**). Nevertheless, even without pronounced phenological shifts, the severe drought in 2022 could have indirectly influenced growth in 2023 by depleting nutrient reserves and reducing cambial activity (Zlobin, 2024).

These growth dynamics underscore the critical role of water availability in regulating xylem production. During drought conditions, cambial activity is often downregulated, limiting cell division and elongation due to reduced turgor pressure and decreased carbohydrate allocation to the cambium (Wang et al., 2024). Conversely, under wetter conditions, increased water availability stimulates cambial activity, promoting more extensive cell division and elongation, which leads to the autumn growth peak (Campelo et al., 2021; De Micco et al., 2016). The timing and intensity of rainfall are thus pivotal in determining the amplitude of the autumn growth peak, highlighting the sensitivity of xylem formation to moisture variability.

### 4.2 Impacts of extreme climate on xylem structure

Extreme climatic events, such as severe droughts and heavy rainfall, can induce plastic adjustments in wood formation dynamics, leading to changes in xylem structure (Zlobin, 2022; Fonti et al., 2010). Variability in precipitation directly affects rates of cell enlargement and wall thickening, which ultimately shape xylem morphology (Silvestro et al., 2023; Cuny et al., 2014). These structural modifications reflect the trees’ adaptive responses to environmental stress. However, prolonged droughts or intense rainfall can disrupt typical growth patterns and result in structural anomalies in the xylem (Niccoli et al., 2024; Zhang et al., 2020).

In 2022, a severe drought from August to October reduced soil moisture and increased vapor pressure deficit (VPD), which severely restricted cambial activity (**Table 2**; Du et al., 2024; Salomón et al., 2022; He et al., 2024). Consequently, both *C. lanceolata* and *C. fortunei* exhibited a unimodal growth pattern, with xylem production limited to the early growing season. This drought-induced cessation of cell division prevented the formation of an autumn growth peak and led to significantly reduced xylem production (**Figure 2**). In contrast, the substantial rainfall events in late July and early September 2023 reactivated cambial activity and accelerated cell division. The subsequent increase in water availability during latewood formation facilitated cell enlargement, leading to the development of L-IADFs. L-IADFs, characterized by large lumen diameters and thin cell walls, are crucial for enhancing water storage and improving xylem water transport efficiency under humid conditions (Niccoli et al., 2024; Bing et al., 2022).

L-IADFs formation is a key adaptive response that optimizes water transport under fluctuating moisture conditions. By enlarging the xylem lumina, trees increase hydraulic conductivity, thereby reducing resistance to water flow and ensuring efficient water transport. This structural adaptation is essential for sustaining metabolic functions during periods of high humidity and enables trees to cope effectively with moisture variability. However, cellular structure is influenced not only by variations in moisture but also by the integrated interplay of plant physiological mechanisms. For instance, water potential, cell turgor, and plant hormones (e.g., auxin and cytokinins) work together to regulate xylem cell expansion and wall thickening. Ren et al. (2015) demonstrated that reduced soil water potential diminishes cell turgor, thereby inhibiting radial expansion in vascular tissues. Buttò et al. (2020) showed that exogenous application of cytokinins under moderate drought conditions can partially restore cambial activity, highlighting the intricate hormonal interplay under water stress. Meanwhile, Niccoli et al. (2024) found that rapid rehydration following a drought can trigger compensatory growth spurts, culminating in the formation of anomalous density bands (L-IADFs). Consistent with these findings, the sustained rainfall during late summer and autumn of 2023 in our study not only reactivated cambial division but also provided the conditions necessary for accelerated cell enlargement, ultimately producing L-IADFs.

### 4.3 Bimodal growth dynamics do not guarantee the occurrence of L-IADFs

While both 2021 and 2023 exhibited bimodal growth patterns, L-IADFs were not observed in 2021 (**Figure 5**; **Supplementary Figure S1**), which contrasts with findings from previous studies (Niccoli et al., 2024; Campelo et al., 2021; Liu et al., 2018). This discrepancy suggests that bimodal growth dynamics do not automatically result in L-IADFs formation, as these two phenomena are not directly correlated. Timely water replenishment during late summer and early autumn can reactivate cambial activity, promoting xylem cell division and leading to a second growth peak (Camarero et al., 2010). However, although cambial activity resumed in 2021, the rate of cell enlargement was insufficient to promote L-IADFs formation, indicating that the presence of an autumn growth peak does not always lead to L-IADFs.

In contrast, the abundant and sustained precipitation during late summer and autumn in 2023 not only stimulated cambial cell division but also significantly accelerated xylem cell enlargement, resulting in the formation of L-IADFs (**Figure 4**; **Figure 5**). These results emphasize that a bimodal growth pattern alone is insufficient to predict the formation of L-IADFs. Rather, the combination of sufficient moisture availability and the rate of cell expansion is crucial for L-IADFs formation. Therefore, while bimodal growth dynamics indicate cambial reactivation, the structural modifications necessary for L-IADFs formation depend on both precise water replenishment and the tree’s physiological capacity for rapid cell enlargement. Nevertheless, our observations are limited to just three years. Consequently, pinpointing a definitive water threshold for L-IADFs formation remains challenging. Future work involving longer-term observations and controlled experiments—coupled with real-time monitoring of physiological indicators—may help clarify whether such a threshold exists. This approach would not only deepen our understanding of L-IADFs formation but also offer empirical support for more robust modeling efforts.

Understanding these adaptive mechanisms is crucial for predicting the long-term impacts of climate change on forest ecosystems. Prolonged droughts or unpredictable rainfall patterns can disrupt traditional growth cycles, leading to structural anomalies in xylem formation. These anomalies may result in increased fluctuations in wood density, which can negatively affect xylem hydraulic efficiency and carbon allocation within trees—critical processes for forest health and productivity. Frequent occurrences of IADFs may indicate instability in wood formation driven by environmental stress, which could signal a decrease in forest ecosystem resilience. This instability reflects the diminished capacity of trees to maintain stable growth patterns and adapt to fluctuating environmental conditions (Fang and Zhang, 2024; Battipaglia et al., 2010). Future research should explore the physiological thresholds beyond which forest species are unable to recover from drought stress or tolerate extreme climatic events. Insights into these thresholds will be vital for developing forest management strategies that enhance the resilience of subtropical forests under the pressures of global climate change.

## 5 Conclusions

This study highlights the crucial role of late-summer precipitation in shaping the growth dynamics and xylem structure of *Cunninghamia lanceolata* and *Cryptomeria fortunei* in humid subtropical China. Our findings demonstrate that sufficient late-summer rainfall promotes a bimodal growth pattern, whereas the autumn drought of 2022 shortened the growing season by approximately two months, eliminating the secondary growth peak and resulting in an unusual unimodal growth pattern. In contrast, continuous late-summer rainfall in 2023 accelerated the enlargement rate of latewood cells, leading to the formation of L-IADFs, characterized by large lumens and thin cell walls. Importantly, our results show that the occurrence of a secondary growth peak does not necessarily lead to the formation of L-IADFs, as observed in 2021. Extreme climatic events, including prolonged droughts and heavy rainfall, can disrupt typical growth patterns and induce structural anomalies in xylem formation. These disruptions highlight the sensitivity of trees to moisture variability, which can impair hydraulic efficiency and affect overall growth stability. Understanding the complex responses of tree growth and xylem structure to fluctuating precipitation regimes is essential for predicting forest dynamics and developing strategies to protect forest health. As climate models forecast increasing precipitation variability, future research should focus on the long-term effects of extreme climate events on wood formation and forest growth. Insights from such research will be critical for developing adaptive forest management strategies aimed at enhancing the resilience of subtropical forests to the impacts of global climate change.

## Data Availability

The original contributions presented in the study are included in the article/**Supplementary Material**. Further inquiries can be directed to the corresponding authors.

## Supplementary material

Supplementary Figure 1 Wood anatomy in tree-rings of *Cu. lanceolata* **(A–C)** and *Cr. fortunei* **(D–F)** sampled at the Gushan Mountain during 2021 **(A, D)**, 2022 **(B, E)** and 2023 **(C, F)** in humid subtropical China. Arrows indicate the L-IADF.
