# Supplementary material for: Linking growth dynamics and intra-annual density fluctuations to late-summer precipitation in humid subtropical China
Source: Front Plant Sci. 2025 Jun 25;16:1568882. doi: 10.3389/fpls.2025.1568882 (PMC12239755; doi:10.3389/fpls.2025.1568882)
Supplement: Supplementary Figure 1 — Wood anatomy in tree-rings of Cu. lanceolata (A–C) and Cr. fortunei (D–F) sampled at the Gushan Mountain during 2021 (A, D), 2022 (B, E) and 2023 (C, F) in humid subtropical China. Arrows indicate the L-IADF. [file DataSheet1.docx]

**Supplementary Materials**

**Linking growth dynamics and intra-annual density fluctuations to late-summer precipitation in the humid subtropical China**

Chunsong Wang^1,2,#^, Zhuangpeng Zheng^3,#^, Jiani Gao^1,2^, Feifei Zhou^1,2*^

Sergio Rossi^4^, Keyan Fang^1,2*^

1 Institute of Geography, Fujian Normal University, Fuzhou 350007, China

2 Key Laboratory of Humid Subtropical Eco-Geographical Process (Ministry of Education), College of Geographical Sciences, Fujian Normal University, Fuzhou 350007, China

3 School of Tourismand Historical Culture, Zhaoqing University; Zhaoqing 526061, China

4 Département des Sciences Fondamentales, Université du Québec à Chicoutimi, 555, boulevard de l’Université Chicoutimi, Chicoutimi, QC, G7H2B1, Canada

^#^ Chunsong Wang and Zhuangpeng Zheng contributed equally to this work

*Corresponding authors: [kfang@fjnu.edu.cn](mailto:kfang@fjnu.edu.cn), [zhouff1987@126.com](mailto:zhouff1987@126.com)


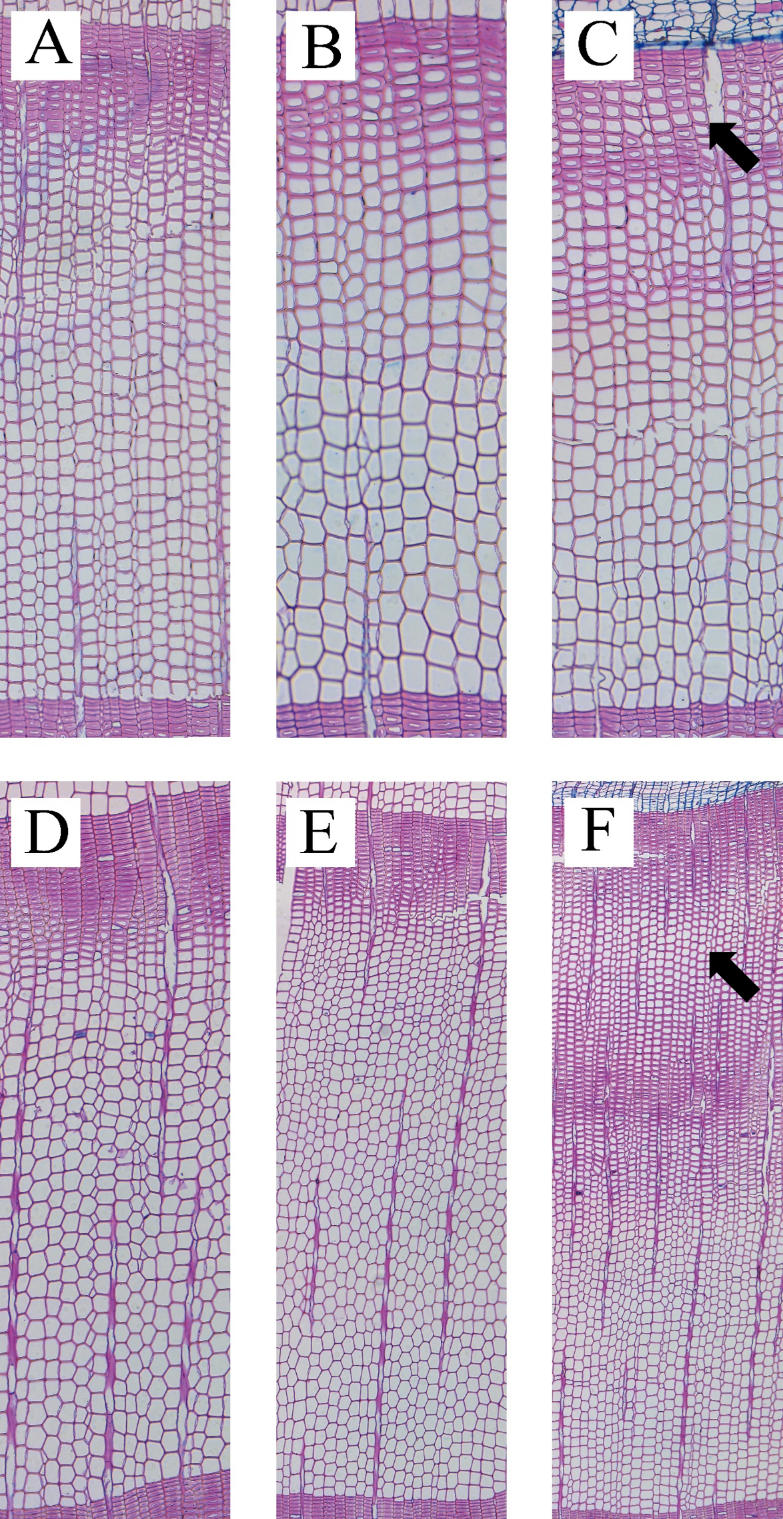


**Supplementary Fig.1** Wood anatomy in tree-rings of *Cu. lanceolata* (A, B, C) and *Cr. fortunei* (D, E, F) sampled at the Gushan Mountain during 2021 (A, D), 2022 (B, E) and 2023 (C, F) in humid subtropical China. Arrows indicate the L-IADF.
